# 

**Published:** 1878-07

**Authors:** 


EDITED BY JULIUS F. MINER, M. D.

Professor of Special and Clinical Surgery in the Buffalo Medical College; Surgeon to the Buffalo General Hospital; Surgeon to Buffalo Hospital of the Sisters of Charity, etc., etc.

EDWARD N. BRUSH, M. D.

Physician to Buffalo Hospital of the Sisters of Charity, etc., etc.

W. W. MINER, A. M., M. D.

Surgeon to Buffalo Hospital of the Sisters of Charity, etc., etc.

BUFFALO.
PUBLISHED FOR THE EDITORS.

1878.

